# 1-Ethyl-4-[1-(1-phenyl­ethyl­idene)hydrazin-2-yl­idene]-3,4-dihydro-1*H*-2λ^6^,1-benzothia­zine-2,2-dione

**DOI:** 10.1107/S1600536812022982

**Published:** 2012-05-31

**Authors:** Muhammad Shafiq, Islam Ullah Khan, Muhammad Nadeem Arshad, Iftikhar Hussain Bukhari, Ejaz

**Affiliations:** a Department of Chemistry, Government College University, Faisalabad 38040, Pakistan; b Materials Chemistry Laboratory, Department of Chemistry, GC University, Lahore 54000, Pakistan; c Department of Chemistry, University of Gujrat, Gujrat 50781, Pakistan

## Abstract

In the title compound, C_18_H_19_N_3_O_2_S, the thia­zine ring adopts an envelope conformation, with the S atom displaced by 0.732 (1) Å from the other atoms of the ring. The phenyl ring is oriented at a dihedral angle of 79.33 (7)° with respect to the fused benzene ring. The conformations about the two double bonds in the *R*_2_C=N—N=C(CH_3_)Ar grouping are *Z* and *E*, respectively. In the crystal, inversion dimers linked by pairs of C—H⋯O inter­actions generate *R*_2_^2^(8) and *R*_2_^2^(12) loops, as parts of infinite chains along the *a*-axis direction.

## Related literature

For related structures and further synthetic details, see: Shafiq *et al.* (2011*a* ▶,*b* ▶). For ring puckering parameters, see: Cremer & Pople (1975 ▶).
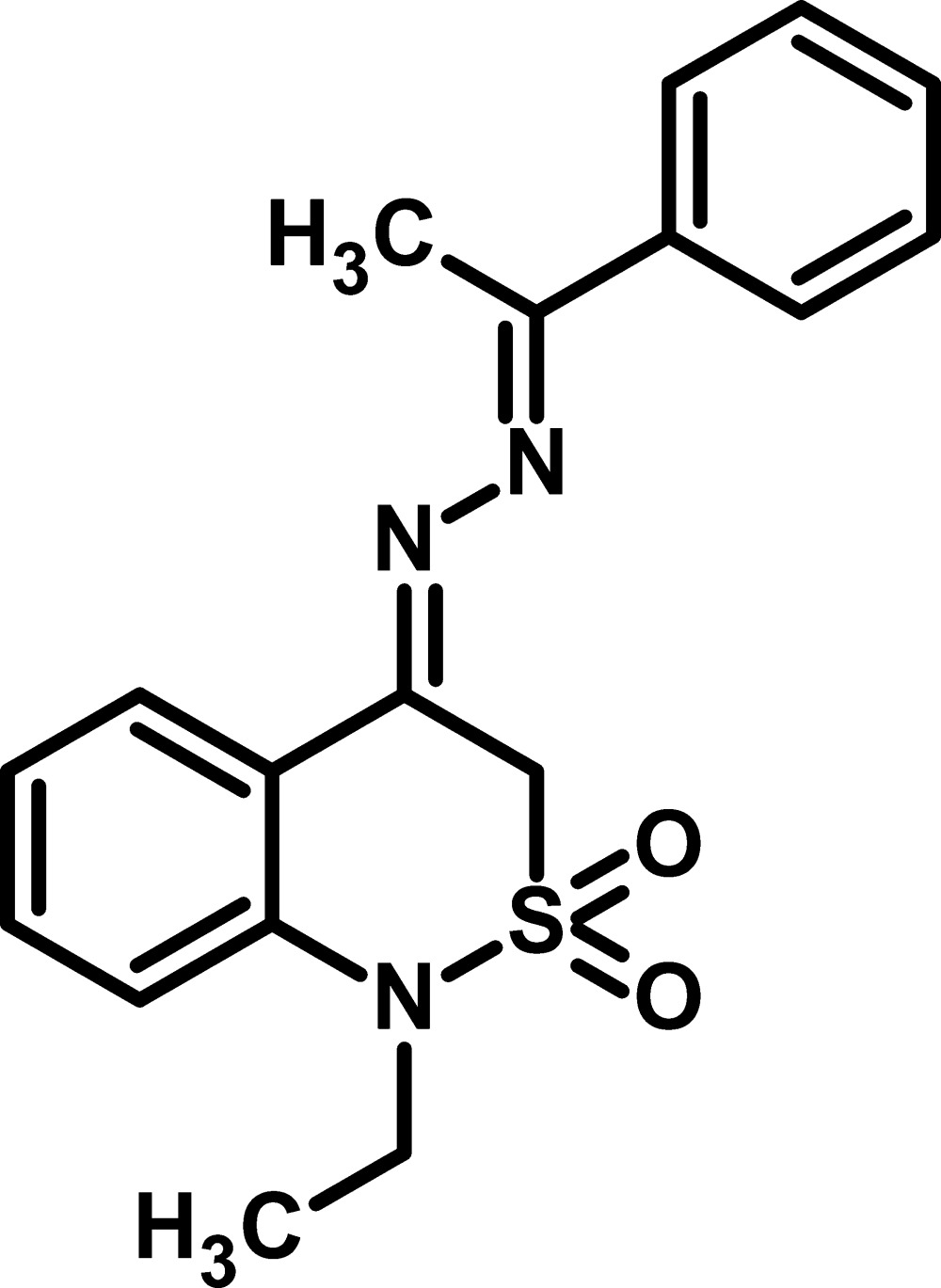


## Experimental

### 

#### Crystal data

C_18_H_19_N_3_O_2_S
*M* *_r_* = 341.42
Monoclinic, 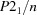

*a* = 9.7278 (3) Å
*b* = 12.4327 (3) Å
*c* = 14.2607 (4) Å
β = 100.725 (1)°
*V* = 1694.60 (8) Å^3^
*Z* = 4
Mo *K*α radiation
μ = 0.21 mm^−1^
*T* = 296 K
0.41 × 0.08 × 0.06 mm

#### Data collection

Bruker Kappa APEXII CCD diffractometer
Absorption correction: multi-scan (*SADABS*; Bruker, 2007 ▶) *T*_min_ = 0.920, *T*_max_ = 0.988
16049 measured reflections
4129 independent reflections
2887 reflections with *I* > 2σ(*I*)
*R*_int_ = 0.035

#### Refinement

*R*[*F* ^2^ > 2σ(*F* ^2^)] = 0.046
*wR*(*F* ^2^) = 0.151
*S* = 1.00
4127 reflections
219 parameters
H-atom parameters constrained
Δρ_max_ = 0.28 e Å^−3^
Δρ_min_ = −0.32 e Å^−3^

### 

Data collection: *APEX2* (Bruker, 2007 ▶); cell refinement: *SAINT* (Bruker, 2007 ▶); data reduction: *SAINT*; program(s) used to solve structure: *SHELXS97* (Sheldrick, 2008 ▶); program(s) used to refine structure: *SHELXL97* (Sheldrick, 2008 ▶); molecular graphics: *PLATON* (Spek, 2009 ▶); software used to prepare material for publication: *WinGX* (Farrugia, 1999 ▶) and *PLATON*.

## Supplementary Material

Crystal structure: contains datablock(s) I, global. DOI: 10.1107/S1600536812022982/hb6798sup1.cif

Structure factors: contains datablock(s) I. DOI: 10.1107/S1600536812022982/hb6798Isup2.hkl

Supplementary material file. DOI: 10.1107/S1600536812022982/hb6798Isup3.cml

Additional supplementary materials:  crystallographic information; 3D view; checkCIF report

## Figures and Tables

**Table 1:** Hydrogen-bond geometry (Å, °)

*D*—H⋯*A*	*D*—H	H⋯*A*	*D*⋯*A*	*D*—H⋯*A*
C8—H8*A*⋯O1^i^	0.97	2.43	3.390 (2)	170
C16—H16*B*⋯O2^ii^	0.97	2.52	3.481 (2)	171

Symmetry codes: (i) 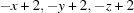; (ii) 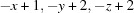.

**Fig. 1.**
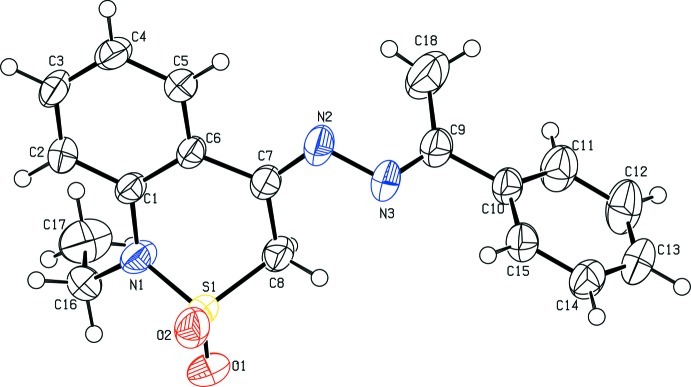
The molecular structure of (I) with 50% displacement ellipsoids.

**Fig. 2.**
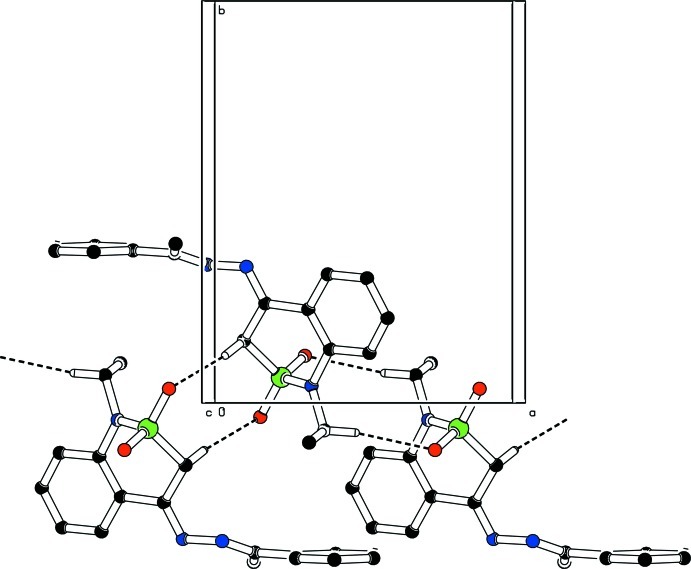
Unit cell packing showes the dimers formed through C—H···O hydrogen bonds, drawn using dashed lines.

## supplementary crystallographic information

### Comment

As part of our ongoing studies of benzothiazines, we now describe the title compound, which is related to 4-hydrazinylidene-1-ethyl-3*H*-2λ^6^,1-benzothiazine-2,2-dione and 6-bromo-1-methyl-4-[2-(4-methylbenzylidene) hydrazinylidene]-3*H*-2λ^6^,1-benzothiazine-2,2-dione. The aromatic and thiazine rings are oriented at dihedral angle of 10.31 (9)° and thiazine ring adopted sofa shape with r. m. s. deviavtion of about 0.2213 (12)° with the maximum deviation of from the S1 (0.3686 (10)Å) & N1 (0.2640 (11)Å). Thiazine ring showes total ring puckering amplitude QT = 0.5430 Å with (θ) = 55.02 ° (π) = 358.2638 ° (Cremer & Pople, 1975). Both the oxygen atoms of SO_2_ group are involved in accepting C—H···O type weak intermolecular hydrogen bonding interaction and produce two ring motifs represented as *R*_2_^2^(8) & *R*_2_^2^(12) (Table. 1, Fig. 2).

### Experimental

4-Hydrazinylidene-1- methyl-3*H*-2λ^6^,1-benzothiazine-2,2-dione (Shafiq *et al.*, 2011*a*) was subjected to react with acetophenone according to literature procedure (Shafiq *et al.*, 2011*b*). The product obtained was then recrystalized in ethylacetate under slow evaporation to obtain suitable crystal for diffraction studies.

### Refinement

All the C—H and H-atoms were positioned with idealized geometry with C—H = 0.93 Å for aromatic, C—H = 0.96 Å for methyl group & C—H = 0.97 Å for methylene and were refined using a riding model with *U*_iso_(H) = 1.2 *U*_eq_(C) for aromatic & methylene and *U*_iso_(H) = 1.5 *U*_eq_(C) for methyl carbon atoms. The two reflections (0 0 2) & (0 1 1) were omitted in final refinement.

### Crystal data

**Table:**

C_18_H_19_N_3_O_2_S	*F*(000) = 720
*M**_r_* = 341.42	*D*_x_ = 1.338 Mg m^−^^3^
Monoclinic, *P*2_1_/*n*	Mo *K*α radiation, λ = 0.71073 Å
Hall symbol: -P 2yn	Cell parameters from 4849 reflections
*a* = 9.7278 (3) Å	θ = 2.4–26.1°
*b* = 12.4327 (3) Å	µ = 0.21 mm^−^^1^
*c* = 14.2607 (4) Å	*T* = 296 K
β = 100.725 (1)°	Needle, colorless
*V* = 1694.60 (8) Å^3^	0.41 × 0.08 × 0.06 mm
*Z* = 4	

### Data collection

**Table:**

Bruker Kappa APEXII CCD diffractometer	4129 independent reflections
Radiation source: fine-focus sealed tube	2887 reflections with *I* > 2σ(*I*)
Graphite monochromator	*R*_int_ = 0.035
φ and ω scans	θ_max_ = 28.3°, θ_min_ = 2.2°
Absorption correction: multi-scan (*SADABS*; Bruker, 2007)	*h* = −12→12
*T*_min_ = 0.920, *T*_max_ = 0.988	*k* = −16→16
16049 measured reflections	*l* = −19→16

### Refinement

**Table:**

Refinement on *F*^2^	Primary atom site location: structure-invariant direct methods
Least-squares matrix: full	Secondary atom site location: difference Fourier map
*R*[*F*^2^ > 2σ(*F*^2^)] = 0.046	Hydrogen site location: inferred from neighbouring sites
*wR*(*F*^2^) = 0.151	H-atom parameters constrained
*S* = 1.00	*w* = 1/[σ^2^(*F*_o_^2^) + (0.0895*P*)^2^ + 0.2125*P*] where *P* = (*F*_o_^2^ + 2*F*_c_^2^)/3
4127 reflections	(Δ/σ)_max_ = 0.002
219 parameters	Δρ_max_ = 0.28 e Å^−^^3^
0 restraints	Δρ_min_ = −0.32 e Å^−^^3^

### Special details

**Table:**

Geometry. All esds (except the esd in the dihedral angle between two l.s. planes) are estimated using the full covariance matrix. The cell esds are taken into account individually in the estimation of esds in distances, angles and torsion angles; correlations between esds in cell parameters are only used when they are defined by crystal symmetry. An approximate (isotropic) treatment of cell esds is used for estimating esds involving l.s. planes.
Refinement. Refinement of F^2^ against ALL reflections. The weighted R-factor wR and goodness of fit S are based on F^2^, conventional R-factors R are based on F, with F set to zero for negative F^2^. The threshold expression of F^2^ > 2sigma(F^2^) is used only for calculating R-factors(gt) etc. and is not relevant to the choice of reflections for refinement. R-factors based on F^2^ are statistically about twice as large as those based on F, and R- factors based on ALL data will be even larger.

### Fractional atomic coordinates and isotropic or equivalent isotropic displacement parameters (Å^2^)

**Table:**

	*x*	*y*	*z*	*U*_iso_*/*U*_eq_	
S1	0.78767 (5)	0.93644 (4)	1.05780 (3)	0.04426 (17)	
O1	0.85670 (16)	1.03503 (13)	1.08838 (12)	0.0650 (4)	
O2	0.71460 (14)	0.88277 (12)	1.12183 (10)	0.0557 (4)	
N1	0.68626 (18)	0.95785 (12)	0.95522 (11)	0.0477 (4)	
N2	0.89644 (17)	0.66151 (14)	0.96946 (13)	0.0556 (4)	
N3	1.02548 (17)	0.65607 (14)	1.03083 (13)	0.0558 (5)	
C1	0.62452 (18)	0.86638 (14)	0.90335 (12)	0.0367 (4)	
C2	0.49509 (19)	0.87561 (16)	0.84347 (14)	0.0458 (5)	
H2	0.4487	0.9414	0.8380	0.055*	
C3	0.43489 (19)	0.78866 (17)	0.79221 (14)	0.0496 (5)	
H3	0.3487	0.7963	0.7518	0.059*	
C4	0.50112 (19)	0.69015 (17)	0.80018 (14)	0.0477 (5)	
H4	0.4594	0.6311	0.7662	0.057*	
C5	0.62979 (19)	0.68026 (15)	0.85904 (13)	0.0428 (4)	
H5	0.6744	0.6138	0.8645	0.051*	
C6	0.69472 (16)	0.76729 (14)	0.91056 (12)	0.0355 (4)	
C7	0.83541 (17)	0.75244 (15)	0.96944 (13)	0.0401 (4)	
C8	0.90722 (18)	0.84518 (16)	1.02506 (14)	0.0473 (5)	
H8A	0.9653	0.8820	0.9869	0.057*	
H8B	0.9677	0.8183	1.0821	0.057*	
C9	1.1295 (2)	0.62777 (15)	0.99497 (15)	0.0497 (5)	
C10	1.26558 (19)	0.62074 (14)	1.06248 (13)	0.0428 (4)	
C11	1.3907 (2)	0.6245 (2)	1.03087 (17)	0.0634 (6)	
H11	1.3910	0.6287	0.9658	0.076*	
C12	1.5168 (2)	0.6219 (2)	1.09532 (19)	0.0724 (7)	
H12	1.6007	0.6258	1.0731	0.087*	
C13	1.5185 (2)	0.61366 (18)	1.19091 (17)	0.0589 (6)	
H13	1.6030	0.6108	1.2338	0.071*	
C14	1.3945 (2)	0.60965 (17)	1.22303 (15)	0.0534 (5)	
H14	1.3951	0.6043	1.2882	0.064*	
C15	1.2685 (2)	0.61348 (15)	1.15988 (14)	0.0471 (5)	
H15	1.1851	0.6112	1.1828	0.057*	
C16	0.6440 (2)	1.06836 (15)	0.92463 (15)	0.0509 (5)	
H16A	0.6842	1.1183	0.9745	0.061*	
H16B	0.5430	1.0742	0.9159	0.061*	
C17	0.6891 (3)	1.0986 (2)	0.83489 (19)	0.0788 (8)	
H17A	0.7893	1.0963	0.8440	0.118*	
H17B	0.6572	1.1701	0.8170	0.118*	
H17C	0.6500	1.0492	0.7854	0.118*	
C18	1.1213 (3)	0.6043 (3)	0.89159 (19)	0.0970 (10)	
H18A	1.0284	0.5811	0.8643	0.146*	
H18B	1.1868	0.5485	0.8844	0.146*	
H18C	1.1436	0.6681	0.8596	0.146*	

### Atomic displacement parameters (Å^2^)

**Table:**

	*U*^11^	*U*^22^	*U*^33^	*U*^12^	*U*^13^	*U*^23^
S1	0.0375 (3)	0.0554 (3)	0.0365 (3)	0.00157 (19)	−0.00216 (19)	−0.0074 (2)
O1	0.0602 (9)	0.0675 (10)	0.0606 (10)	−0.0076 (7)	−0.0063 (8)	−0.0235 (8)
O2	0.0478 (8)	0.0793 (10)	0.0402 (8)	0.0117 (7)	0.0085 (6)	0.0057 (7)
N1	0.0578 (10)	0.0388 (8)	0.0392 (9)	0.0005 (7)	−0.0099 (7)	0.0001 (7)
N2	0.0431 (9)	0.0559 (10)	0.0599 (11)	0.0109 (7)	−0.0106 (8)	0.0007 (9)
N3	0.0418 (9)	0.0636 (11)	0.0548 (11)	0.0136 (8)	−0.0097 (8)	0.0023 (9)
C1	0.0347 (9)	0.0442 (10)	0.0292 (8)	0.0000 (7)	0.0009 (7)	−0.0002 (7)
C2	0.0389 (10)	0.0519 (11)	0.0433 (10)	0.0099 (8)	−0.0009 (8)	0.0002 (9)
C3	0.0335 (9)	0.0670 (13)	0.0425 (11)	0.0018 (8)	−0.0077 (8)	−0.0021 (10)
C4	0.0447 (10)	0.0531 (11)	0.0416 (11)	−0.0072 (8)	−0.0021 (8)	−0.0084 (9)
C5	0.0426 (9)	0.0443 (10)	0.0396 (10)	0.0031 (7)	0.0023 (8)	−0.0025 (8)
C6	0.0307 (8)	0.0441 (10)	0.0300 (8)	0.0010 (7)	0.0014 (6)	0.0022 (7)
C7	0.0332 (9)	0.0498 (10)	0.0358 (9)	0.0016 (7)	0.0027 (7)	0.0014 (8)
C8	0.0306 (9)	0.0653 (12)	0.0437 (11)	0.0009 (8)	0.0010 (8)	−0.0070 (9)
C9	0.0460 (11)	0.0508 (11)	0.0484 (12)	0.0076 (8)	−0.0011 (9)	0.0052 (9)
C10	0.0396 (10)	0.0426 (10)	0.0435 (10)	0.0069 (7)	0.0009 (8)	0.0049 (8)
C11	0.0523 (13)	0.0906 (17)	0.0473 (12)	0.0097 (11)	0.0093 (10)	0.0146 (12)
C12	0.0398 (11)	0.1026 (19)	0.0750 (17)	0.0039 (11)	0.0114 (11)	0.0222 (14)
C13	0.0417 (11)	0.0669 (14)	0.0612 (14)	−0.0007 (9)	−0.0082 (10)	0.0135 (11)
C14	0.0525 (12)	0.0610 (12)	0.0429 (11)	0.0054 (9)	−0.0007 (9)	0.0061 (10)
C15	0.0419 (10)	0.0497 (11)	0.0487 (11)	0.0068 (8)	0.0057 (9)	0.0053 (9)
C16	0.0536 (12)	0.0411 (10)	0.0550 (12)	0.0094 (8)	0.0020 (10)	−0.0072 (9)
C17	0.111 (2)	0.0568 (14)	0.0637 (15)	−0.0098 (14)	0.0021 (15)	0.0094 (12)
C18	0.0702 (17)	0.163 (3)	0.0505 (15)	0.0186 (19)	−0.0083 (13)	−0.0103 (18)

### Geometric parameters (Å, º)

**Table:**

S1—O2	1.4232 (15)	C9—C10	1.487 (3)
S1—O1	1.4264 (15)	C9—C18	1.490 (3)
S1—N1	1.6270 (16)	C10—C11	1.376 (3)
S1—C8	1.7493 (19)	C10—C15	1.387 (3)
N1—C1	1.427 (2)	C11—C12	1.390 (3)
N1—C16	1.477 (2)	C11—H11	0.9300
N2—C7	1.277 (2)	C12—C13	1.364 (3)
N2—N3	1.392 (2)	C12—H12	0.9300
N3—C9	1.266 (3)	C13—C14	1.368 (3)
C1—C2	1.388 (2)	C13—H13	0.9300
C1—C6	1.403 (2)	C14—C15	1.380 (3)
C2—C3	1.373 (3)	C14—H14	0.9300
C2—H2	0.9300	C15—H15	0.9300
C3—C4	1.379 (3)	C16—C17	1.477 (3)
C3—H3	0.9300	C16—H16A	0.9700
C4—C5	1.377 (2)	C16—H16B	0.9700
C4—H4	0.9300	C17—H17A	0.9600
C5—C6	1.392 (3)	C17—H17B	0.9600
C5—H5	0.9300	C17—H17C	0.9600
C6—C7	1.478 (2)	C18—H18A	0.9600
C7—C8	1.497 (3)	C18—H18B	0.9600
C8—H8A	0.9700	C18—H18C	0.9600
C8—H8B	0.9700		
			
O2—S1—O1	118.02 (10)	N3—C9—C18	123.7 (2)
O2—S1—N1	111.22 (9)	C10—C9—C18	120.4 (2)
O1—S1—N1	107.84 (9)	C11—C10—C15	118.41 (18)
O2—S1—C8	107.66 (9)	C11—C10—C9	121.41 (19)
O1—S1—C8	109.90 (9)	C15—C10—C9	120.15 (18)
N1—S1—C8	100.84 (9)	C10—C11—C12	120.6 (2)
C1—N1—C16	121.41 (15)	C10—C11—H11	119.7
C1—N1—S1	117.52 (12)	C12—C11—H11	119.7
C16—N1—S1	120.46 (13)	C13—C12—C11	120.5 (2)
C7—N2—N3	114.08 (16)	C13—C12—H12	119.7
C9—N3—N2	117.05 (18)	C11—C12—H12	119.7
C2—C1—C6	119.40 (16)	C12—C13—C14	119.3 (2)
C2—C1—N1	119.94 (16)	C12—C13—H13	120.3
C6—C1—N1	120.64 (15)	C14—C13—H13	120.3
C3—C2—C1	120.69 (17)	C13—C14—C15	120.7 (2)
C3—C2—H2	119.7	C13—C14—H14	119.6
C1—C2—H2	119.7	C15—C14—H14	119.6
C2—C3—C4	120.59 (17)	C14—C15—C10	120.42 (19)
C2—C3—H3	119.7	C14—C15—H15	119.8
C4—C3—H3	119.7	C10—C15—H15	119.8
C5—C4—C3	119.17 (17)	N1—C16—C17	112.46 (18)
C5—C4—H4	120.4	N1—C16—H16A	109.1
C3—C4—H4	120.4	C17—C16—H16A	109.1
C4—C5—C6	121.62 (17)	N1—C16—H16B	109.1
C4—C5—H5	119.2	C17—C16—H16B	109.1
C6—C5—H5	119.2	H16A—C16—H16B	107.8
C5—C6—C1	118.50 (15)	C16—C17—H17A	109.5
C5—C6—C7	118.95 (15)	C16—C17—H17B	109.5
C1—C6—C7	122.54 (15)	H17A—C17—H17B	109.5
N2—C7—C6	119.48 (16)	C16—C17—H17C	109.5
N2—C7—C8	120.71 (16)	H17A—C17—H17C	109.5
C6—C7—C8	119.76 (15)	H17B—C17—H17C	109.5
C7—C8—S1	111.91 (12)	C9—C18—H18A	109.5
C7—C8—H8A	109.2	C9—C18—H18B	109.5
S1—C8—H8A	109.2	H18A—C18—H18B	109.5
C7—C8—H8B	109.2	C9—C18—H18C	109.5
S1—C8—H8B	109.2	H18A—C18—H18C	109.5
H8A—C8—H8B	107.9	H18B—C18—H18C	109.5
N3—C9—C10	115.90 (18)		
			
O2—S1—N1—C1	−59.42 (16)	C1—C6—C7—N2	−176.73 (18)
O1—S1—N1—C1	169.72 (14)	C5—C6—C7—C8	179.76 (17)
C8—S1—N1—C1	54.52 (16)	C1—C6—C7—C8	0.7 (3)
O2—S1—N1—C16	111.72 (17)	N2—C7—C8—S1	−153.81 (16)
O1—S1—N1—C16	−19.14 (19)	C6—C7—C8—S1	28.8 (2)
C8—S1—N1—C16	−134.34 (16)	O2—S1—C8—C7	64.98 (16)
C7—N2—N3—C9	−125.1 (2)	O1—S1—C8—C7	−165.27 (14)
C16—N1—C1—C2	−21.2 (3)	N1—S1—C8—C7	−51.62 (16)
S1—N1—C1—C2	149.88 (15)	N2—N3—C9—C10	−178.94 (16)
C16—N1—C1—C6	157.43 (18)	N2—N3—C9—C18	2.2 (3)
S1—N1—C1—C6	−31.5 (2)	N3—C9—C10—C11	−159.4 (2)
C6—C1—C2—C3	0.7 (3)	C18—C9—C10—C11	19.5 (3)
N1—C1—C2—C3	179.27 (18)	N3—C9—C10—C15	18.7 (3)
C1—C2—C3—C4	0.8 (3)	C18—C9—C10—C15	−162.4 (2)
C2—C3—C4—C5	−1.1 (3)	C15—C10—C11—C12	−0.5 (3)
C3—C4—C5—C6	−0.1 (3)	C9—C10—C11—C12	177.6 (2)
C4—C5—C6—C1	1.5 (3)	C10—C11—C12—C13	1.2 (4)
C4—C5—C6—C7	−177.54 (17)	C11—C12—C13—C14	−1.0 (4)
C2—C1—C6—C5	−1.8 (3)	C12—C13—C14—C15	0.2 (3)
N1—C1—C6—C5	179.60 (17)	C13—C14—C15—C10	0.4 (3)
C2—C1—C6—C7	177.25 (16)	C11—C10—C15—C14	−0.3 (3)
N1—C1—C6—C7	−1.4 (3)	C9—C10—C15—C14	−178.37 (18)
N3—N2—C7—C6	−177.29 (16)	C1—N1—C16—C17	−69.1 (3)
N3—N2—C7—C8	5.3 (3)	S1—N1—C16—C17	120.06 (19)
C5—C6—C7—N2	2.3 (3)		

### Hydrogen-bond geometry (Å, º)

**Table:**

*D*—H···*A*	*D*—H	H···*A*	*D*···*A*	*D*—H···*A*
C8—H8*A*···O1^i^	0.97	2.43	3.390 (2)	170
C16—H16*B*···O2^ii^	0.97	2.52	3.481 (2)	171

Symmetry codes: (i) −*x*+2, −*y*+2, −*z*+2; (ii) −*x*+1, −*y*+2, −*z*+2.
